# Subcutaneous Immunoglobulin Replacement Therapy with Hizentra® is Safe and Effective in Children Less Than 5 Years of Age

**DOI:** 10.1007/s10875-015-0190-0

**Published:** 2015-08-17

**Authors:** Niraj C. Patel, Joel L. Gallagher, Hans D. Ochs, Thomas Prescott Atkinson, Justin Wahlstrom, Morna Dorsey, Francisco A. Bonilla, Jennifer Heimall, Lisa Kobrynski, David Morris, Elie Haddad

**Affiliations:** 1 Department of Pediatrics, Section of Infectious Disease and Immunology, Levine Children’s Hospital at Carolinas Medical Center, PO Box 32861, Charlotte, NC 28203 USA; 2 Department of Pediatrics, Seattle Children’s Research Institute and University of Washington, Seattle, WA USA; 3 Department of Pediatrics, University of Alabama at Birmingham, Birmingham, AL USA; 4 Present Address: Department of Pediatrics, University of California, San Francisco, CA USA; 5 Department of Pediatrics, University of South Florida, Tampa, FL USA; 6 Department of Pediatrics, Boston Children’s Hospital, Boston, MA USA; 7 Department of Pediatrics, Children’s Hospital of Philadelphia, Philadelphia, PA USA; 8 Department of Pediatrics, Emory University, Atlanta, GA USA; 9 WebbWrites, Durham, NC USA; 10 Department of Pediatrics, Department of Microbiology, Infectiology, and Immunology, University of Montreal, Québec, Canada; 11 Present Address: Medical College of Wisconsin, Milwaukee, WI USA

**Keywords:** Immunoglobulin, Subcutaneous, Immunodeficiency, Children, Safety, Efficacy

## Abstract

**Background:**

Hizentra® (IGSC 20 %) is a 20 % liquid IgG product approved for subcutaneous administration in adults and children 2 years of age and older who have primary immunodeficiency disease (PIDD). There is limited information about the use of IGSC 20 % in very young children including those less than 5 years of age.

**Methods:**

A retrospective chart review involved 88 PIDD infants and children less than 5 years of age who received Hizentra®.

**Results:**

The mean age at the start of Hizentra® was 34 months (range 2 to 59 months). IGSC 20 % was administered weekly to 86 infants (two additional infants received twice weekly and three times weekly infusions, respectively) and included an average of 63 infusions (range 6–182) for an observation period up to 45.5 months. Infusion by manual delivery occurred in 15 patients. The mean dose was 674 mg/kg/4 weeks. The mean IgG level was 942 mg/dL while on IGSC 20 %, compared to a mean trough IgG level of 794 mg/dL (*p* < 0.0001) during intravenous or subcutaneous IgG administration prior to IGSC 20 %. Average infusion time was 47 (range 5–120) minutes, and the median number of infusion sites was 2 (range 1–4). Local reactions were mostly mild and observed in 36/88 (41 %) children. No serious adverse events were reported. A significant increase in weight percentile (7 % ± 19.2, *p* = 0.0012) among subjects was observed during IGSC 20 % administration. The rate of serious bacterial infections was 0.067 per patient-year while receiving IGSC 20 %, similar to previously reported efficacy studies.

**Conclusions:**

Hizentra® is effective in preventing infections, and is well tolerated in children less than age 5 years.

## Introduction

Primary immunodeficiency diseases (PIDD) confer increased risk of recurrent respiratory, skin, and gastrointestinal infections [1–5]. Intravenous IgG replacement therapy (IVIG), indicated for patients with antibody deficiency, is commonly given every 3–4 weeks [6–10]. Subcutaneously-administered IgG (SCIG) has increasingly been used as alternative replacement therapy for PIDD patients [11–15]. Because of low infusion volume and ease of administration, SCIG is well suited for children.

Hizentra® (IGSC 20 %), a 20 % liquid SCIG formulation, is approved for adults and children greater than 2 years of age with PIDD [16–21]. The higher concentration allows a lower infusion volume and shorter infusion time. Experience in children less than 5 years of age is limited [22, 23].

We evaluated the efficacy and safety of IGSC 20 % in children less than age 5 years. Because growth restriction in early childhood has been associated with a high infectious disease burden [24–26], weight and height percentiles during IGSC 20 % infusions were assessed.

## Methods

### Patients

A retrospective review of Hizentra® replacement therapy was performed in 88 infants and children under age 5 years from 9 North American centers. Patients were included if they received >1 dose of IGSC 20 % prior to age 5 years, regardless of previous replacement. Patients with protein losing conditions or concomitant treatment with IVIG while on IGSC 20 % were excluded. Study data were managed using Research Electronic Data Capture (REDCap) program hosted at Carolinas Medical Center [27]. The study was approved by the Institutional Review Board at all participating institutions.

### Efficacy and Safety Assessments

Study assessments included rates of serious bacterial infections (SBIs) (bacteremia/sepsis, meningitis, pneumonias, osteomyelitis, septic arthritis, visceral abscesses), non-serious infections, adverse events (AEs), local reactions, volume and duration of infusions, number of infusion sites, and serum IgG levels [28]. Rate of infusions was assessed for pump or manual delivery, and a cut-off value of >20 ml/h was used for rapid delivery based on previously published studies [13, 23, 29].

### Growth Parameter Assessments

Weight and body length at the start and end of treatment were recorded. Only patients with values at both time points were included. Results were provided for all patients, 4 subgroups described by gender and age at start of Hizentra® (females ≤24 months, females >24–59 months, males ≤24 months, and males >24–59 months), and those who received hematopoietic stem cell transplantation (HSCT, *n* = 15) or gene therapy (*n* = 1). The difference in mean weight and length percentile was evaluated using a t-test. The analysis of covariance was used to test for sex differences.

## Results

### Study Population

Demographics and underlying PIDD diagnoses are shown (Table 1). Fifty-three of the 88 infants were male and the most common race was white. The predominant PIDD conditions included T-cell or combined immunodeficiency (*n* = 26), primary antibody deficiency (*n* = 19), and isolated hypogammaglobulinemia with repeated infections (*n* = 25). The mean age at the start of Hizentra® was 34 (range 2–59) months.

**Table 1:** Demographics, Characteristics, and Diagnosis for 88 PIDD Infants and Children at the Start of Hizentra®

Patient characteristic	34 Patients, Aged 0 to <2 Years, No. (%)	54 Patients, Aged 2 to 5 Years, No. (%)	88 Patients Total, No. (%)
Gender, n (%)			
Male	23 (26)	30 (34)	53 (60)
Female	11 (13)	24 (27)	35 (40)
Race, n (%)			
White	29 (33)	41 (46)	70 (80)
Black or African American	4 (5)	4 (5)	8 (9)
Asian	1 (1)	3 (3)	4 (4)
Other	0 (0)	6 (7)	6 (7)
Age and Growth Parameters			
Age, months, mean (range)	11 (2–23)	44 (25–59)	34 (2–59)
Height, cm, mean (range)	70.0 (50.6–91)	94.5 (76.3–116.5)	84.3 (50.6–116.5)
Weight, kg, mean (range)	8.7 (4.1–15.5)	15.0 (9.3–28.4)	12.5 (4.1–28.4)
Diagnosis, n (%)			
T-cell and combined immunodeficiencies			
Severe combined immunodeficiency	6 (6.8)	8 (9)	14 (16)
Hyperimmunoglobulin M syndrome	0 (0)	1 (1)	1 (1)
22q11 deletion	0 (0)	5 (5.7)	5 (5.7)
Ataxia-telangiectasia	0 (0)	2 (2)	2 (2)
Wiscott-Aldrich syndrome	1 (1)	0 (0)	1 (1)
Combined immunodeficiency, unspecified	3 (3.4)	0 (0)	3 (3.4)
Total	10 (11.4)	16 (18.2)	26 (29.6)
Predominantly antibody deficiencies			
Common variable immunodeficiency	0 (0)	5* (6)	5* (6)
X-linked agammaglobulinemia	7 (8)	4 (4.5)	11 (12.5)
Immunoglobulin G subclass deficiency	0 (0)	2 (2)	2 (2)
Autosomal recessive agammaglobulinemia	0 (0)	1 (1)	1 (1)
Total	7 (8)	12 (14)	19 (22)
Disorders of innate immunity			
NF-κB essential modulator (NEMO)	2 (2)	1 (1)	3 (3)
Total	2 (2)	1 (1)	3 (3)
Other			
Hypogammaglobulinemia with specific antibody deficiency	1 (1)	7 (8)	8 (9)
Isolated Hypogammaglobulinemia with repeated infections	11 (12.5)	14 (16)	25 (28.4)
Transient hypogammaglobulinemia of infancy	2 (2)	2 (2)	4 (4.5)
Hyperimmunoglobulin E syndrome (STAT3)	0 (0)	2 (2)	2 (2)
Netherton syndrome	1 (1)	0 (0)	1 (1)
Total	15 (17)	25 (28)	40 (45)

### Infusion Parameters

Most infants received weekly IGSC 20 % therapy (*n* = 86) for 1.5–45.5 (average 14.25) months, one was infused twice weekly (by pump) for 10.25 months, and one was infused three times weekly (by manual delivery) for 8 months (Table 2). Seventy-two of 88 patients received prior Ig therapy including 48 patients who received IVIG, and 24 patients who received SCIG (Vivaglobin®, 16 %, CSL Behring). Sixteen patients (18 %) were naïve to Ig therapy prior to starting Hizentra®.

**Table 2:** Summary of 5572 Hizentra® Infusions for 88 Children

	34 Patients, Aged 0 to <2 Years	54 Patients, Aged 2 to <5 Years	88 Patients Total
Median dosage, mg/kg/4 weeks (range)	753 (400–2000)	602 (260–2000)	674 (260–2000)
Mean number of infusions (range)	69 (10–182)	60 (6–137)	63 (6–182)
Mean infusion time, minutes (range)	49 (5–120)	46 (5–120)	47 (5–120)
Median number of infusion sites (range)	2.0 (1–3)	2.0 (1–4)	2.0 (1–4)
Mean volume per patient, mL (range)	8.3 (4–13)	11.2 (5–38)	10.1 (4–38)
Mean volume per site, mL (range)	4.5 (1.5–13.5)	5.7 (2.5–20.6)	5.2 (1.5–20.6)

**Fig. 1:**
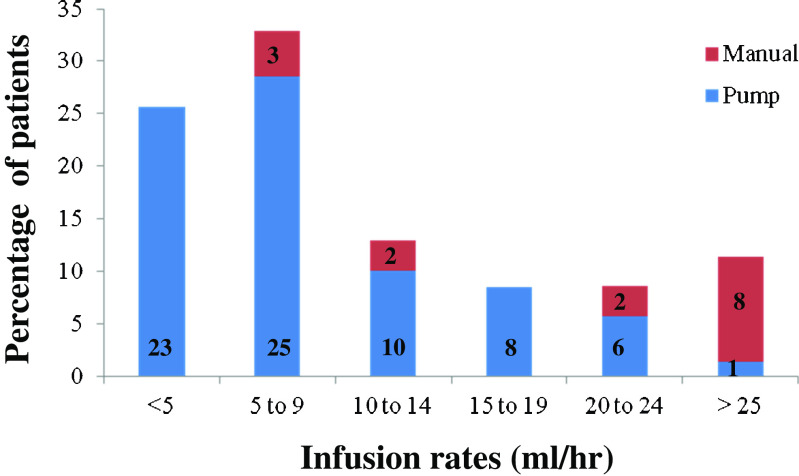
Infusion rates for 88 pediatric patients receiving Hizentra® by pump or manual delivery. Data are presented as the percentage of patients and number of patients within each category

Median cumulative monthly IGSC 20 % dosage was 674 mg/kg, compared to a monthly dosage of 552 mg/kg of Ig prior to IGSC 20 % treatment. Infusion rates (Fig. 1) of <20 ml/h occurred in 66/73 (90 %) (median 5.9 ml/h, range 1.3–18.9) subjects infused by pump compared to 5/15 (33 %) (median 10 ml/h, range 7.5–12) subjects infused by manual delivery, respectively. Rates >20 ml/h occurred in 7 of 73 (10 %) (median 20.6 ml/h, range 20–39.8) subjects infused by pump compared to 10/15 (67 %) (median 29.7 ml/h, range 22.3–89.8) infused by manual delivery.

Of 46 patients receiving IVIG before being changed to IGSC 20 %, 20 underwent dose adjustment by a factor of 1.53 (as recommended by the FDA) while 12 patients received the same IVIG and IGSC 20 % dose and 9 patients received a weekly dose lower than calculated for IVIG; 5 patients received dosing above the recommended 1.53 adjustment factor. Mean IgG levels were higher for the 88 patients receiving IGSC 20 % (942 mg/dL) compared to the 72 patients receiving IVIG or SCIG therapy prior to being switched to IGSC 20 % (794 mg/dL) (*p* < 0.0001). Thirty-three patients had IgG levels for review during IVIG and subsequent IGSC 20 % administration: the mean IgG trough level on IVIG was 720 mg/dL while mean IgG level on IGSC 20 % was 943 mg/dL (*p* < 0.0001, paired t-test).

For 72 patients who transitioned to Hizentra® from another IgG therapy, reasons for change included difficult IV access (*n* = 26), family/patient preference (*n* = 31), discontinuation of prior product (Vivaglobin®) (*n* = 22), side effects (*n* = 5), transportation problems (*n* = 3), more reliable dosing (*n* = 1), and discontinuation of a peripherally-inserted central catheter (*n* = 1).

Sites of administration included thigh (58 %), abdomen (24 %), thigh and abdomen (17 %), and arm and thigh (1 %). Hizentra® was administered by pump (*n* = 73) or manual delivery (*n* = 15). Manual delivery was most often accomplished using a 23-, 25-, or 27-guage butterfly needle set and the mean volume per site was 4.7 mL (range 2.0–7.5 mL). Needle length was reported in 55 patients and the most frequently used size was 6 mm (80 %), followed by 4 mm (15 %) and 9 mm (5 %). The median number of administration sites was 2 (range 1–4 sites). After a short instruction phase, Hizentra® was administered at home by a parent or caregiver administered in all but two patients who received administration by a home care nurse or other provider.

### Efficacy

The incidence of SBIs and non-serious infections per patient-year (Table 3), respectively, were 0.067 and 0.29 during Hizentra® administration, 0.22 and 0.37 prior to Hizentra® administration (including 72 patients previously treated with Ig), and 0.25 and 0.40 prior to any Ig therapy.

**Table 3:** Comparison of Number and Rate of Infections Per Patient-Year Occurring During Treatment Prior to and during Hizentra® (N = 88)

	Prior to Hizentra®^a^		Hizentra®	
Infection Type	Total Number of Infections	Rate of Infections Per Patient-Year	Total Number of Infections	Rate of Infections Per Patient-Year
Serious Bacterial Infections				
Bacteremia/sepsis	20	0.09	3	0.03
Bacterial meningitis	2	0.01	0	0
Pneumonia, bacterial	42	0.20	3	0.03
Osteomyelitis/septic arthritis	0	0	1	0.01
Visceral abscess (liver,lung,brain)	3	0.01	0	0
Total	47	0.22	7	0.067
Non-Serious Infections				
Urinary tract infection	8	0.04	0	0
Pneumonia, viral	10	0.05	3	0.03
Ear infection	6	0.03	3	0.03
Gastroenteritis	11	0.05	7	0.06
Abscess/cellulitis	8	0.04	4	0.04
Bronchiolitis/tracheitis	16	0.07	11	0.10
Sinusitis	1	0.005	3	0.03
Viremia (CMV, EBV)	6	0.03	0	0
Lymphadenitis	1	0.005	0	0
Fever	4	0.02	0	0
Other	6^b^	0.03	0	0
Total	77	0.37	31	0.29

^a^Includes 72 patients who received IVIG (*n* = 48) or SCIG other than Hizentra® (*n* = 24)

^b^Other infections included acute respiratory distress syndrome (*n* = 1), necrotizing enterocolitis (*n* = 1), necrotizing fasciitis of the chest wall (*n* = 1), *Staphylococcus aureus* peritonitis (*n* = 1), and infections site-not-specified (Klebsiella, *n* = 1; Enterobacter, *n* = 1)

### Tolerability

Replacement therapy with IGSC 20 % was well tolerated; 41 patients experienced no local AEs (Table 4). Local reactions were mostly mild and observed in 36 children. Upper respiratory tract infections were reported in 15 (17 %) patients. Other AEs included pruritus (*n* = 3), irritability (*n* = 3), and one patient each with bronchitis, pyrexia, sinusitis, and otitis. There were no serious AEs.

**Table 4:** Summary of Adverse Events

Adverse Event	34 Patients, Aged 0 to <2 Years, No. (%)	54 Patients, Aged 2 to <5 Years, No. (%)	88 Patients Total, No. (%)
None	20 (59)	21 (39)	41 (47)
Any adverse event^a^	14 (41)	33 (61)	47 (53)
Local reaction^b^	9 (26)	27 (50)	36 (41)
Upper respiratory tract infection	1 (3)	14 (26)	15 (17)
Pruritus	1 (3)	2 (4.7)	3 (3)
Bronchitis	0 (0)	1 (2)	1 (1)
Pyrexia	0 (0)	1 (2)	1 (1)
Sinusitis	0 (0)	1 (2)	1 (1)
Otitis media	0 (0)	1 (2)	1 (1)
Irritability	3 (9)	0 (0)	3 (3)

^a^Multiple adverse events occurred in some patients

^b^Based on 16 MedDRA preferred terms

IGSC 20 % was discontinued in 14 (15.9 %) patients prior to age 5 years. Reasons for discontinuation included resolved immune deficiency (*n* = 6), physician discontinuation (*n* = 4), parental refusal (*n* = 2), intolerance related to pruritus (*n* = 1), and prolonged hospitalization following renal transplantation (*n* = 1). One patient expired of unrelated causes.

### Growth Parameter Assessments during Hizentra®

**Fig. 2:**
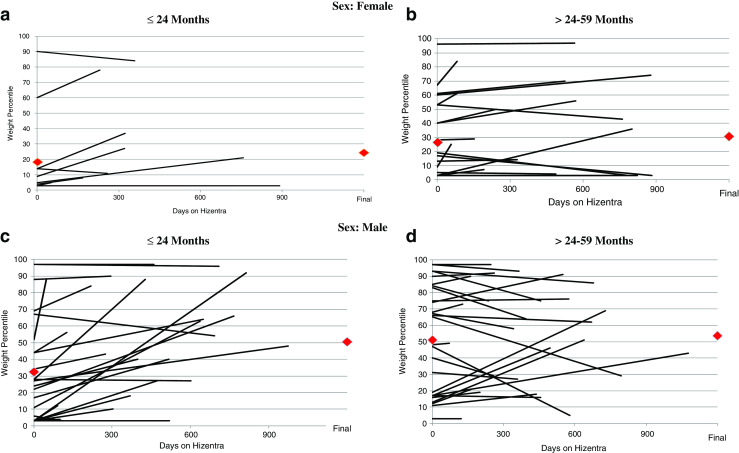
(A-D): Weight percentile measurements are shown at the start and completion of Hizentra® therapy for 86 patients. A and B include females (<=24 and >24–59 months, respectively). C and D similarly show males (<=24 and >24–59 months, respectively). Each line represents 1 patient, and *red diamonds* () represent mean weight percentile at first and last measurement on Hizentra®

**Fig. 3:**
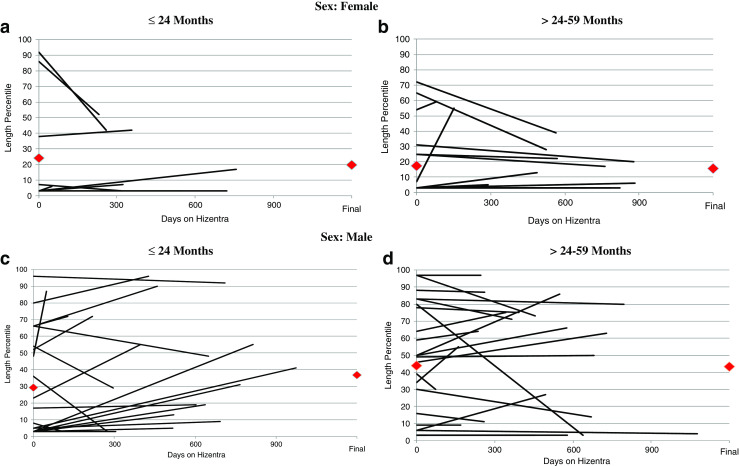
(A-D): Length percentile measurements are shown at the start and completion of Hizentra® therapy for 74 patients. A and B include females (<=24 and >24–59 months, respectively). C and D similarly show males (<=24 and >24–59 months, respectively). Each line represents 1 patient, and *red diamonds* () represent mean length percentile at first and last measurement on Hizentra®

Weight and length percentiles were assessed at the beginning and end of Hizentra® observation period. Among 85 patients for whom weight parameters were available, there was a statistically significant increase in overall weight percentile with a mean increase of 7 % (standard deviation [SD] = 19.2 %) (*p* = 0.0012) (Fig. 2A-D) over the observation period (mean 427 days). Those with an initial weight below 30th percentile saw the most significant gains. The largest increase in weight percentile was in males ≤24 months with a mean of 18 % (SD = 24) (*p* = 0.0009). Among 74 patients for whom length parameters were available, there was an overall increase in length percentile of 2 % (SD = 18), but this did not reach statistical significance (*p* = 0.36) (Fig. 3A-D). The greatest increase in length percentile occurred in males ≤24 months with a mean of 9 % (SD = 21) (*p* = 0.052). For 16 patients who underwent HSCT or gene therapy, smaller increases in growth were seen compared to those who did not (Table 5). The difference between males and females was statistically significant for change in length (*p* = 0.018) but not weight (*p* = 0.099) regardless of age at baseline.

**Table 5:** Comparison of Changes in Weight and Height Percentiles Among Transplanted and Non-transplanted Patients During Treatment with Hizentra®

		Change from Baseline			
		Weight Percentile		Height Percentile	
Group	Transplant* Status	N	Mean (SD)	N	Mean (SD)
Male					
≤ 24 months	Transplant	4	8.0 (6.6)	4	2.3 (27.0)
	No Transplant	20	20.3 (25.2)	18	10.7 (20.0)
	Overall	24	18.2 (23.5)	22	9.2 (20.9)
> 24 months	Transplant	6	−0.8 (26.3)	6	2.5 (9.3)
	No Transplant	22	1.5 (17.7)	18	2.3 (14.2)
	Overall	28	1.0 (19.3)	24	2.3 (12.9)
Female					
≤ 24 months	Transplant	3	19.7 (2.9)	3	−11.3 (20.0)
	No Transplant	8	−2.4 (6.3)	7	−8.1 (19.4)
	Overall	11	3.6 (11.6)	10	−9.1 (18.5)
> 24 months	Transplant	3	−5.7 (9.0)	3	3.3 (5.8)
	No Transplant	19	5.6 (10.7)	15	−2.7 (18.7)
	Overall	22	4.0 (11.0)	18	−1.3 (17.3)

*Included patients who underwent hematopoietic stem cell transplantation (*n* = 15) or gene therapy (*n* = 1)

## Discussion

This is the first large retrospective study to examine efficacy and safety of SCIG in very young children. In 88 children less than 5 years of age, including 34 children less than 2 years of age, Hizentra® was safe and efficacious with an annualized rate of SBIs of 0.067 per patient-year. This rate is similar to those observed in older children and adults [16, 17, 19–21]. Mean IgG levels were higher during IGSC 20 % administration (942 mg/dL) compared to IgG levels in the same cohort during receipt of IVIG (794 mg/dL) (*p* < 0.0001). Higher trough IgG levels are known to be associated with a significant decrease in serious infections for PIDD patients [9]. The higher mean dose of IGSC 20 % (674 mg/kg/4 weeks) compared to IVIG may have been due to the converting factor of 1.53 or higher when switch from IVIG to IGSC 20 %. Higher IgG serum levels (17–22 %) on IGSC 20 % compared to IVIG have been previously reported [16, 17], and with equal dosing 1:1, SCIG will result in 13 % average high serum IgG levels compared to IVIG [18]. However, most immunologists believe it is not necessary to give higher doses of Hizentra® including those patients less than age 5 years.

Hizentra® was well tolerated in PIDD patients. Local reactions were mostly mild. Systemic AEs were infrequent and there were no serious AEs. Only one patient discontinued treatment due to pruritus. Low rates (29 %) of local reactions observed in children less than age 2 years may have been related to lower infusion volumes [16, 17, 21]. Manual administration of IGSC 20 % in 15 (17 %) patients without a pump device was tolerated without difficulty. Mean infusion time (47 min) was less than that reported for IGSC 20 % in adolescents (60 min) and adults (85 min) [17] and among children and adults (76 min) [16]. This was likely related to smaller volumes administered to smaller patients, and to some patients who received rapid infusion by manual delivery.

Weight percentile among 85 subjects increased significantly (7 %, *p* = 0.0012) during the observation period (mean 427 days), and those below the 30th percentile for weight saw the largest gains. Similar observations have been reported in young children during catch-up growth after a period of high infectious disease burden or inadequate dietary intake [24, 25, 30–32]. A smaller increase in length percentile was observed (2 %) in 74 subjects, although this was not statistically significant. Previous observations suggest stunted growth in children requires recovery in weight before resuming linear growth [24, 30–32]. Perhaps a longer observation period in the current study would have seen larger gains in length percentiles. Patients receiving HSCT or gene therapy had smaller gains in growth which may have been related to complications associated with treatment, although this was not assessed. Data from this study support the observation that poor growth seen among PIDD patients, most likely related to prior frequent and severe infections, can be reversed with optimal immunoglobulin replacement.

In summary, Hizentra® was effective in preventing infections in PIDD infants and children less than age 5 years. Infusions were associated with an overall increase in weight and length percentiles. Hizentra® is well tolerated with no serious adverse events and is suitable for immunoglobulin replacement in very young children. Hizentra® may also be well-suited for small pediatric patients in whom venous access is difficult.
